# Middle–Late Triassic sedimentary provenance of the southern Junggar Basin and its link with the post-orogenic tectonic evolution of Central Asia

**DOI:** 10.1038/s41598-021-96455-9

**Published:** 2021-08-23

**Authors:** Jialin Wang, Chaodong Wu, Yue Jiao, Bo Yuan

**Affiliations:** 1 Key Laboratory of Orogenic Belts and Crustal Evolution, Ministry of Education, School of Earth and Space Sciences, Peking University, Beijing, 100871 China; 2 Institute of Oil & Gas, Peking University, Beijing, 100871 China; 3 Research Institute of Exploration and Development, Xinjiang Oilfield Company, PetroChina, Karamay, 834000 Xinjiang China

**Keywords:** Geochemistry, Geodynamics, Tectonics

## Abstract

Due to the unknown Triassic volcanism in the Junggar Basin, the Middle–Late Triassic sedimentary provenance in the southern Junggar Basin (SJB) has long been controversial. Detrital zircon grains from 13 samples of the Middle–Upper Triassic Xiaoquangou Group in the SJB were analyzed using zircon U–Pb geochronology to constrain the provenance of Triassic sedimentary rocks and to further understand their source-to-sink system. Comparison of detrital zircon U–Pb age distributions for 13 samples reveals that the Triassic age populations predominate in sediments of the northern Bogda Mountains, with subordinate in the southern Bogda Mountains, and no or minimal in the North Tianshan (NTS). Coupled with sandstone petrological, sedimentary geochemical and paleocurrent data, the Triassic detrital zircon grains of the Xiaoquangou Group in the SJB were probably input from the Bogda Mountains. As Pennsylvanian and Mississippian zircon grains are mainly derived from the NTS and Central Tianshan (CTS), the provenance of the Xiaoquangou Group includes the NTS, CTS and Bogda Mountains. But the different samples in different sink areas have different provenances, originating from at least four source-to-sink systems. The supply of sediments from the Bogda Mountains started in the Late Triassic, suggesting initial uplift of the Bogda Mountains.

## Introduction

Detrital zircon U–Pb geochronology, petrography, paleocurrent measurements and sandstone petrography techniques are widely exploited for source-to-sink analysis^1–18^. Of these, the rationale for detrital zircon U–Pb geochronology is the similarity of zircon U–Pb age distribution patterns from the source and sink areas^6,14^. However, comparison limitations emerge when direct evidence of related detrital zircon U–Pb ages is missing for potential source areas or when the source areas are poorly preserved^14^. This situation is common for deep-time sediment provenance analysis due to intense denudation in source areas and poor exposure of stratigraphic records^14,19–21^. In recent years, researchers have established provenance analysis tools (e.g., probability density functions, kernel density estimates, multidimensional scaling, and cumulative distribution plots) to study the source signature when its source characteristics are unknown^4,11,22^. Some researchers also prove the effectiveness of these methods for inferring source characteristics, particularly when the source area is poorly preserved^18^.

**Figure 1:**
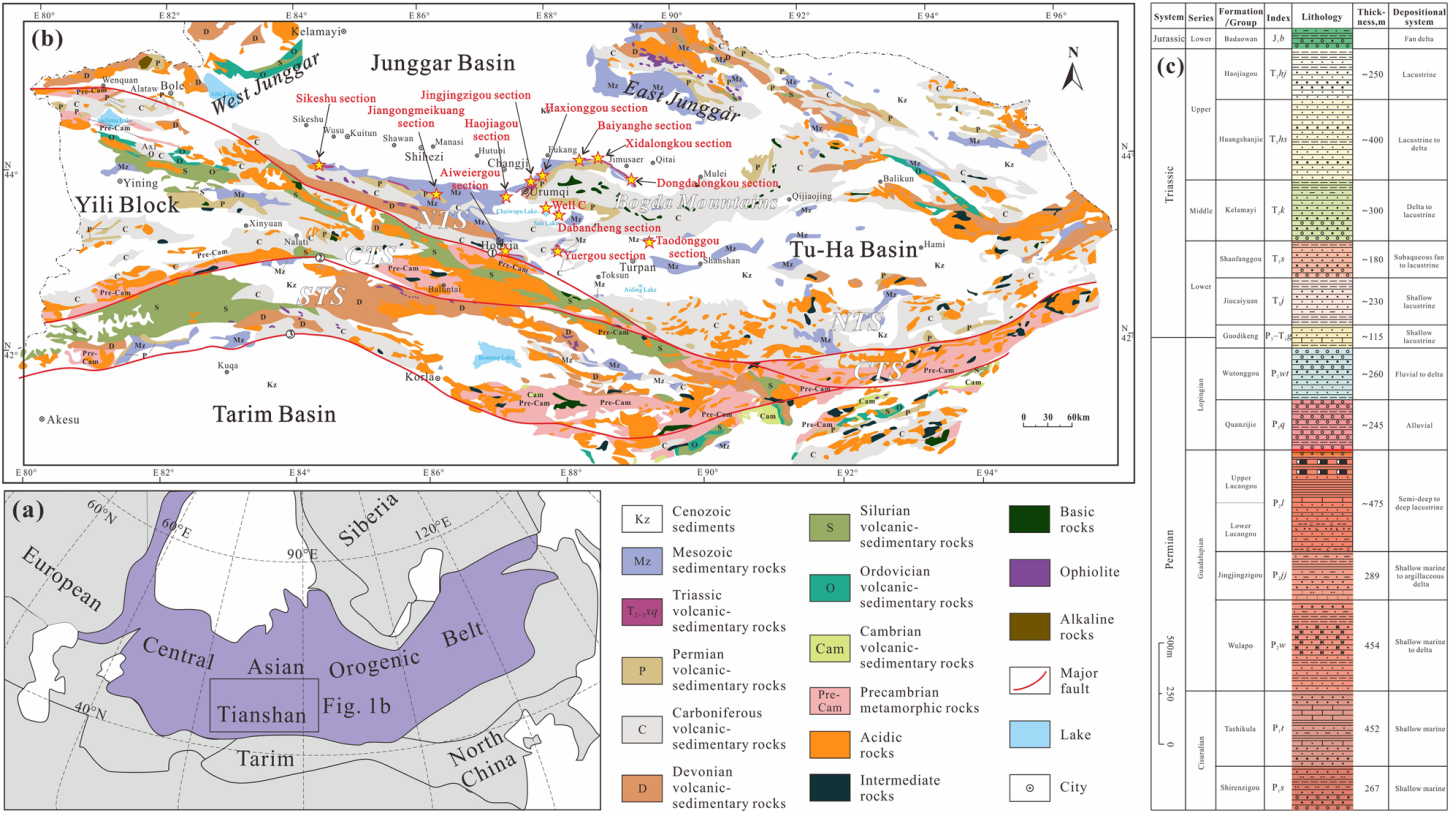
**(a)** Tectonic outlines of the Central Asian Orogenic Belt (modified after^32^). **(b)** Tectonic map of the Tianshan and southern Junggar Basin showing the locations of the studied sections (starred areas; modified after^23^). **(c)** Generalized Permian–Triassic stratigraphic column of the southern Junggar Basin.

In the southern Junggar Basin (SJB) of Central Asian Orogenic Belt (CAOB) (Fig. 1a,b), many detrital zircon grains with Triassic ages are found in sediments of the Middle–Upper Triassic Xiaoquangou Group in the Bogda Mountains^16,17^. These zircon grains show similar ages/epochs to the sedimentary rocks of the Xiaoquangou Group, indicating that the Xiaoquangou Group was deposited with syndepositional magmatism or volcanism. Moreover, the Xiaoquangou Group is subdivided into the Kelamayi Formation (Middle Triassic), Huangshanjie Formation (Upper Triassic) and Haojiagou Formation (Upper Triassic) from bottom to top (Fig. 1c). Compared to underlying strata, the detrital zircon U–Pb age-distribution pattern of the Upper Triassic Haojiagou Formation shows a significant multimodal distribution and presents a dramatic change with the first appearance of zircons from sub-contemporaneous Middle–Late Triassic magmatic sources^16,17^. These phenomena highlight the importance of the provenance of Triassic detrital zircon grains for understanding the source-to-sink system of this area and the tectonic evolution of the Bogda Mountains^16,17,24^. As the presently exposed geology of potential source areas does not include Triassic igneous rocks, previous works have no way to correlate the existence of such detritus with a source area. These Triassic detrital zircon grains, nevertheless, have been interpreted as derivations from the Jiumusaer area of the Bogda Mountains^25^, Harlik Mountains^24^, and Altai Mountains^26^ without robust evidence.

In this contribution, thirteen samples from twelve sections and one well (the Sikeshu, Jiangongmeikuang, and Aiweiergou sections in front of the NTS or western part of the SJB, the Haojiagou, Jingjingzigou, Haxionggou, Baiyanghe, Xidalongkou, and Dongdalongkou sections in the northern Bogda Mountains, the Dabancheng and Yuergou sections, and well C in the southern Bogda Mountains) distributed within the Middle–Upper Triassic Xiaoquangou Group were collected for detrital zircon U–Pb analysis. Then, a combination of probability density plots, kernel density estimates, multidimensional scaling, and cumulative distribution plots of detrital zircon U–Pb ages for thirteen samples were conducted to infer the provenance of Triassic sedimentary rocks in the SJB, especially the provenance of Triassic detrital zircon grains. This study also utilizes other information (e.g., trace element contents) from detrital zircon to further characterize the features of Triassic volcanism. Last, the Triassic source-to-sink relationship of the southern Junggar Basin were established to further constrain the tectonic evolution of the southern CAOB.

## Geological setting

The Junggar Basin is a triangular-shaped sedimentary basin surrounded by the West Junggar systems in the northwest, the East Junggar systems in the northeast, and the Chinese Tianshan and Bogda Mountains in the south^27^ (Fig. 1a,b). The basin and adjacent areas are part of the CAOB formed through the accretion of island arcs, ophiolites, and microcontinental fragments^28–30^. In the Paleozoic, the Junggar Basin, together with the surrounding Bogda rift, West Junggar systems, East Junggar systems, Turpan–Hami Basin, and North Tianshan Ocean, belonged to the southern part of the Paleo–Asian Ocean and was composed of the Ob–Zaisan, Junggar–Balkhash, and Turkestan Oceans from north to south^31^. The SJB (including the present North Tianshan (NTS) and Bogda Mountain areas), represents the southern part of the Junggar–Balkhash Ocean in the CAOB, which is surrounded by the North Tianshan Arc, Central Tianshan Block, East Junggar Arc (Harlik–Dananhu Arc), and West Junggar Remnant Basin^32,33^. In the Mesozoic, the Junggar Basin transformed into an intracontinental basin, which is prevailingly interpreted as a lacustrine system^25,34,35^.

**Figure 2:**
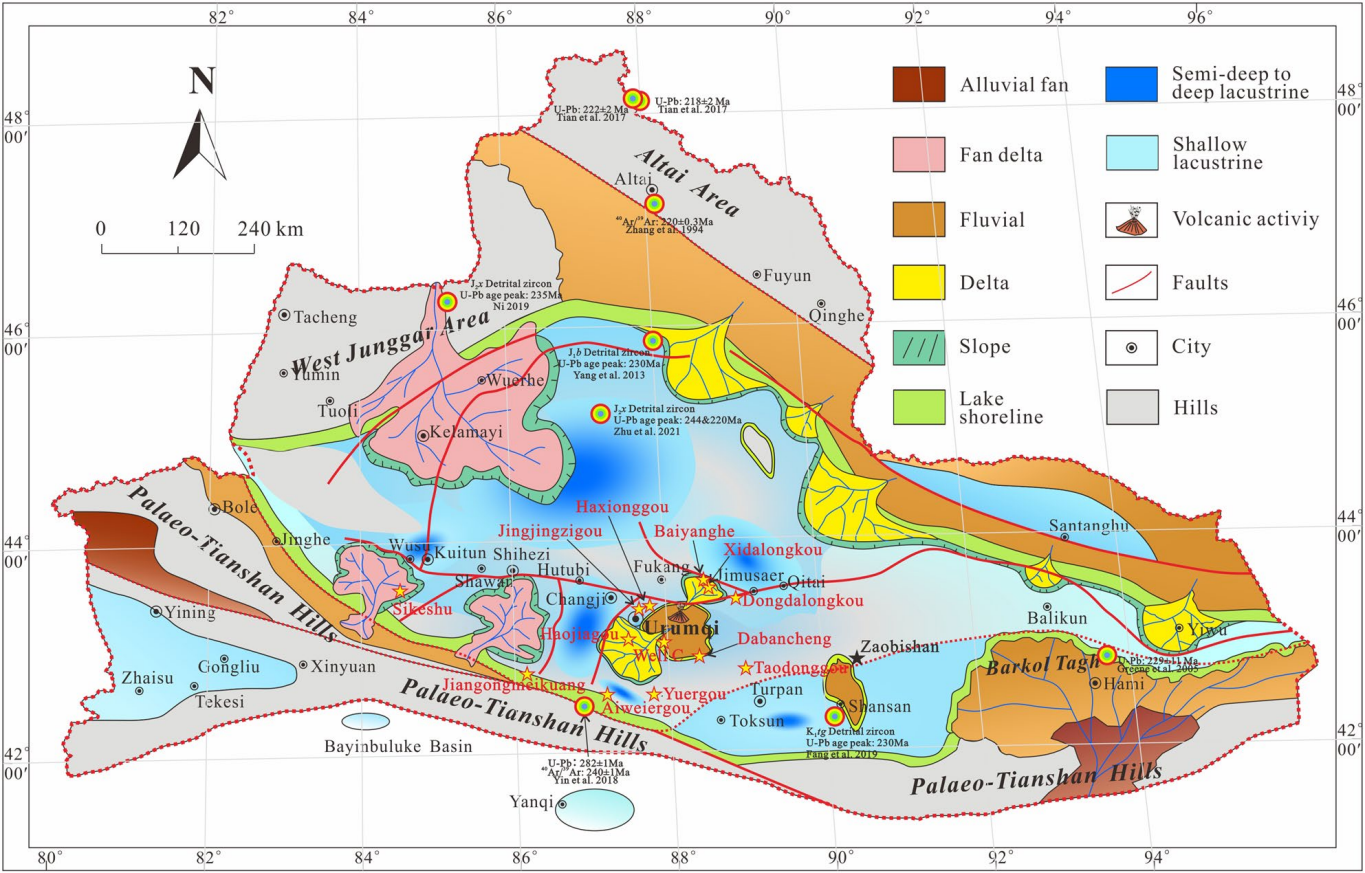
Paleogeographic map of the Junggar Basin and adjacent areas during the Middle–Late Triassic period.

**Figure 3:**
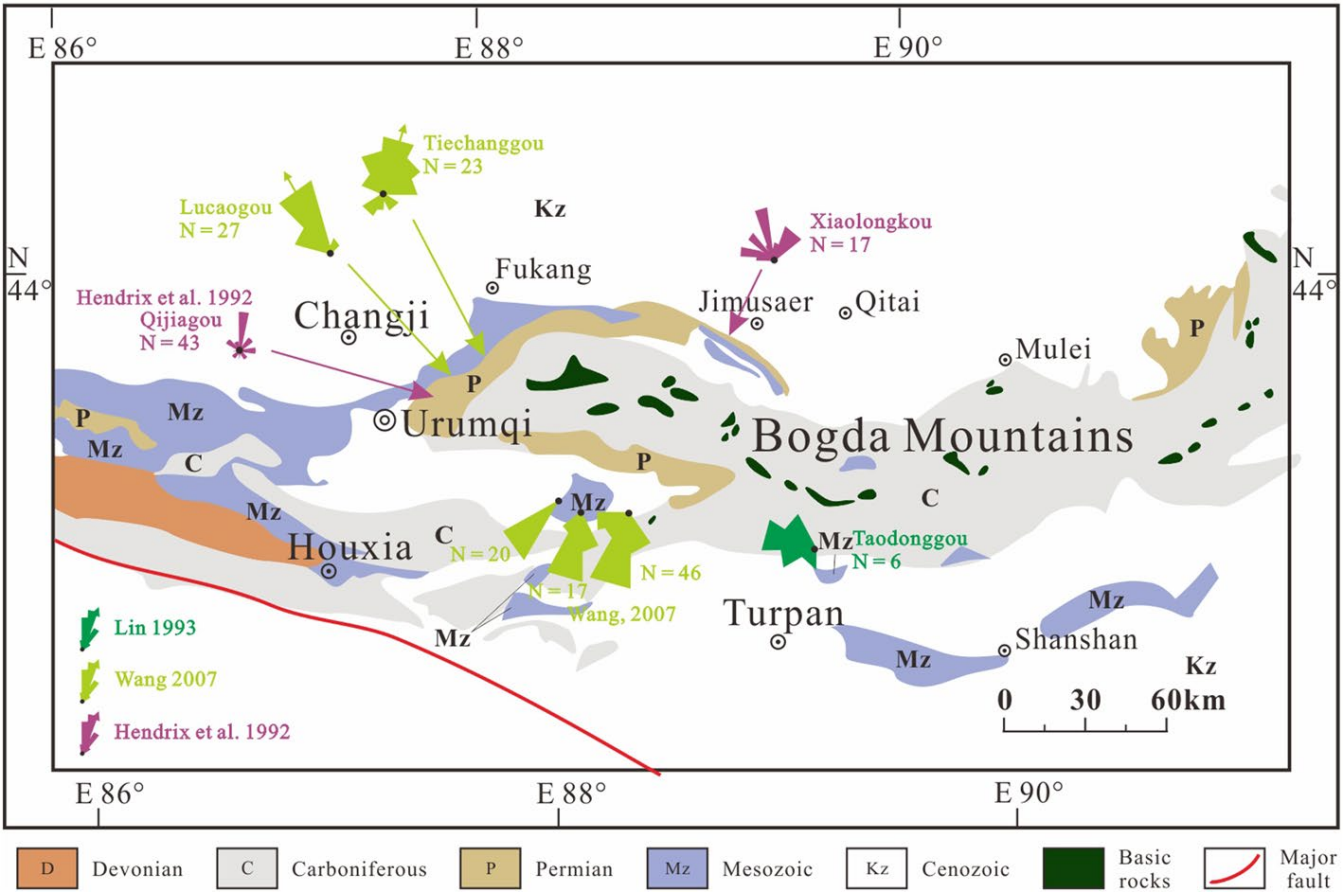
Paleocurrent directions of the Middle–Upper Triassic strata around the Bogda Mountains (modified after^17,42–44^).

The SJB was characterized by rifting (syn-rift stage subsidence controlled by normal faults) in the Permian^16,17,34–39^, followed by depression (post-rift stage subsidence controlled probably by lithospheric cooling) in the Triassic^16,17,34,35,39^. The Permian strata of the SJB are principally composed of clastics, carbonates and volcaniclastics with marine gastropods, brachiopods, corals, crinoids. Sedimentological analysis shows that the Permian sedimentary environments of the SJB comprise marine and continental facies with frequent lateral facies and thickness changes^17,34,35,39,40^. Moreover, various areas display unique sedimentary characteristics, suggesting numerous subbasins composed of a series of half-grabens and/or grabens, such as the Tarlong–Taodonggou half graben or Jimusaer graben^17,35^(Fig. 2). The Triassic strata of the SJB contain mudstone, sandstone and conglomerate with minor coal layers, which are interpreted as fluvial and lacustrine facies sweeping into an intracontinental basin^39^ (Fig. 2). Of these, the Middle–Upper Triassic Xiaoquangou Group is marked by mudstone, argillaceous siltstone and sandy mudstone interbedded with conglomerate. Paleocurrents were mainly northward-directed in the western part of the SJB^12,16,17,41^, while in the eastern part of the SJB, paleocurrents were mainly northward-directed in the northern Bogda Mountains and southwestward-directed in the southern Bogda Mountains^42–44^(Fig. 3).

The NTS is composed of the Devonian to Carboniferous arc-related igneous and volcaniclastic rocks, which have been interpreted as products of the southward subduction of NTS oceanic crust during the late Paleozoic^30,45,46^. The Central Tianshan (CTS) was a continental block with Precambrian basement, and is consist of the Paleozoic northern and southern magmatic belts which generated by the NTS oceanic crust subducting southwards below the CTS Block and the northward subduction of the STS oceanic crust^45^. The tectonostratigraphic units of the CTS comprise Proterozoic basement and early Paleozoic arc-type rocks intruded by late Paleozoic granitoids^32,46^. The Bogda Mountains, like the northern part of the NTS (Fig. 1c), contains mainly the Devonian to Quaternary sedimentary and igneous rocks^45,47^. Among these, Carboniferous bimodal volcanic rocks are widely distributed^48,49^, while the sedimentary rocks primarily include siliciclastics, carbonates, and volcanic breccia, and are characterized by the absence or minor quantities of quartzose sandstone or ophiolite^25^. The East Junggar is dominated by the Ordovician to Permian volcanics, siliciclastic rocks, limestones, and cherts, with an early Paleozoic Zhaheba–Aermantai ophiolite belt extending through its middle part^30,50^. The Silurian–Carboniferous arc magmatism was intense in the Yemaquan area along the southern part of the East Junggar systems, possibly resulting from the northward subduction of Kalamaili oceanic crust^51^. The West Junggar is characterized by Paleozoic volcanic–sedimentary sequences and granitoid intrusions. The Ordovician and Silurian rocks consist of low-grade schists, phyllites, and tuffaceous siliciclastic rocks. The Devonian to early Carboniferous strata is dominated by siliciclastic rocks and felsic–mafic volcanic rocks that mainly formed in marine environments. Above a late Carboniferous depositional hiatus lie Permian terrestrial volcanic and clastic successions^50^.

## Sampling and analytical methods

Medium- to coarse-grained sandstones were collected from twelve sections and one well of the Middle–Upper Triassic Xiaoquangou Group in the SJB for detrital zircon U–Pb analysis (Fig. 1b). The samples, including 12SKS-07, 17JGMK-06, XJ12-06, 17HJG-01, 15HXG-95, 17JJZG-10, 17BYH-61, 16DLK-49, 17DDLK-09, 16TDG-06, 16DBC-04, HX-01, and 15C-33, are from the Sikeshu section (front of the NTS), Jiangongmeikuang section (front of the NTS), Aiweiergou section (front of the NTS), Haojiagou section (northern Bogda Mountains), Haxionggou section (northern Bogda Mountains), Jingjingzigou section (northern Bogda Mountains), Baiyanghe section (northern Bogda Mountains), Xidalongkou section (northern Bogda Mountains), Dongdalongkou section (northern Bogda Mountains), Taodonggou section (southern Bogda Mountains), Dabancheng section (southern Bogda Mountains), Yuergou section (southern Bogda Mountains), and well C (southern Bogda Mountains), respectively. Of these, the data for eight samples, namely, 17JGMK-06 (Middle–Upper Triassic Xiaoquangou Group), 17HJG-01 (Upper Triassic Huangshanjie Formation), 15HXG-95 (Middle–Upper Triassic Xiaoquangou Group), 17JJZG-10 (Middle–Upper Triassic Xiaoquangou Group), 17BYH-61 (Middle Triassic Kelamayi Formation), 17DDLK-09 (Middle Triassic Kelamayi Formation), 16TDG-06 (Upper Triassic Xiaoquangou Group), and 16DBC-04 (Upper Triassic Xiaoquangou Group), are new data and generated in this study. The detrital zircon U–Pb age data for five samples, namely, 12SKS-07 (Upper Triassic Xiaoquangou Group), XJ12-06 (Middle–Upper Triassic Xiaoquangou Group), 15C-33 (Upper Triassic Huangshanjie Formation), 16DLK-49 (Upper Triassic Huangshanjie Formation), and HX-01 (Middle–Upper Triassic Xiaoquangou Group), are from Zhu et al.^52^, Liu et al.^7^, Wang et al.^16^, Wang et al.^17^, and Liu et al.^8^, respectively.

Detrital zircon grains were separated using standard heavy-liquid and magnetic techniques, each sample followed by hand-picking of 250 random zircon grains under a binocular microscope. The grains then underwent cathodoluminescence (CL) imaging and U–Pb isotope analyses by LA–ICP–MS at Rockman Technology Co., Ltd microanalysis lab, Beijing, with detailed procedures following Thompson et al.^53^. The Resolution SE model laser ablation system (Applied Spectra, USA) was equipped with an ATL (ATLEX 300) excimer laser and a Two Volume S155 ablation cell. The laser ablation system was coupled to an Agilent 7900 ICPMS (Agilent, USA). The isotope ratios and element concentrations of the zircon grains were calculated using the Iolite software package^54^. Zircon 91500 and GJ-1 were used as primary and secondary reference materials, respectively. Correction for common lead used the method of Andersen^55^ and the ages were computed using ISOPLOT 3 with uncertainties quoted at the 1σ standard deviation and 95% confidence levels^56^. The probability density plots and kernel density estimates of detrital zircon U–Pb ages were conducted by IsoplotR^22^.

The detrital ages of thirteen samples also collected for classical Multidimensional scaling (MDS) analysis which were conducted by IsoplotR^22^. MDS is a dimension-reducting technique that takes a matrix of pairwise dissimilarities between detrital age distributions as input and produces a configuration of two (or higher-) coordinates as output, so that the Euclidean distance between these coordinates approximates the dissimilarities of the input matrix. Thus, an MDS-configuration serves as a map in which similar samples cluster closely together and dissimilar samples plot far apart^22^.

## Results

### Zircon U–Pb ages

**Figure 4:**
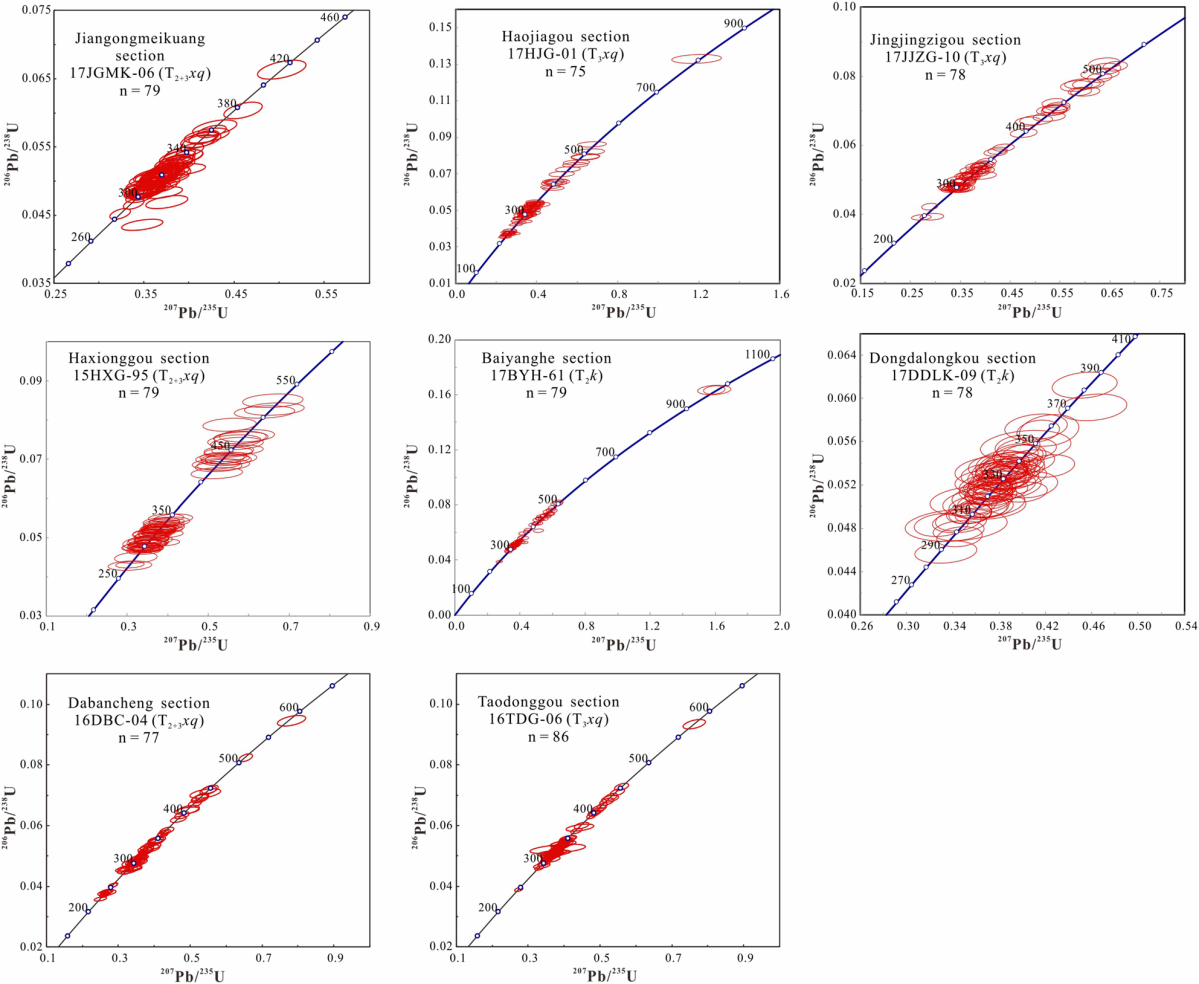
U–Pb concordia diagrams for zircon grains acquired from the Middle–Upper Triassic Xiaoquangou Group in different sections of the southern Junggar Basin.

**Figure 5:**
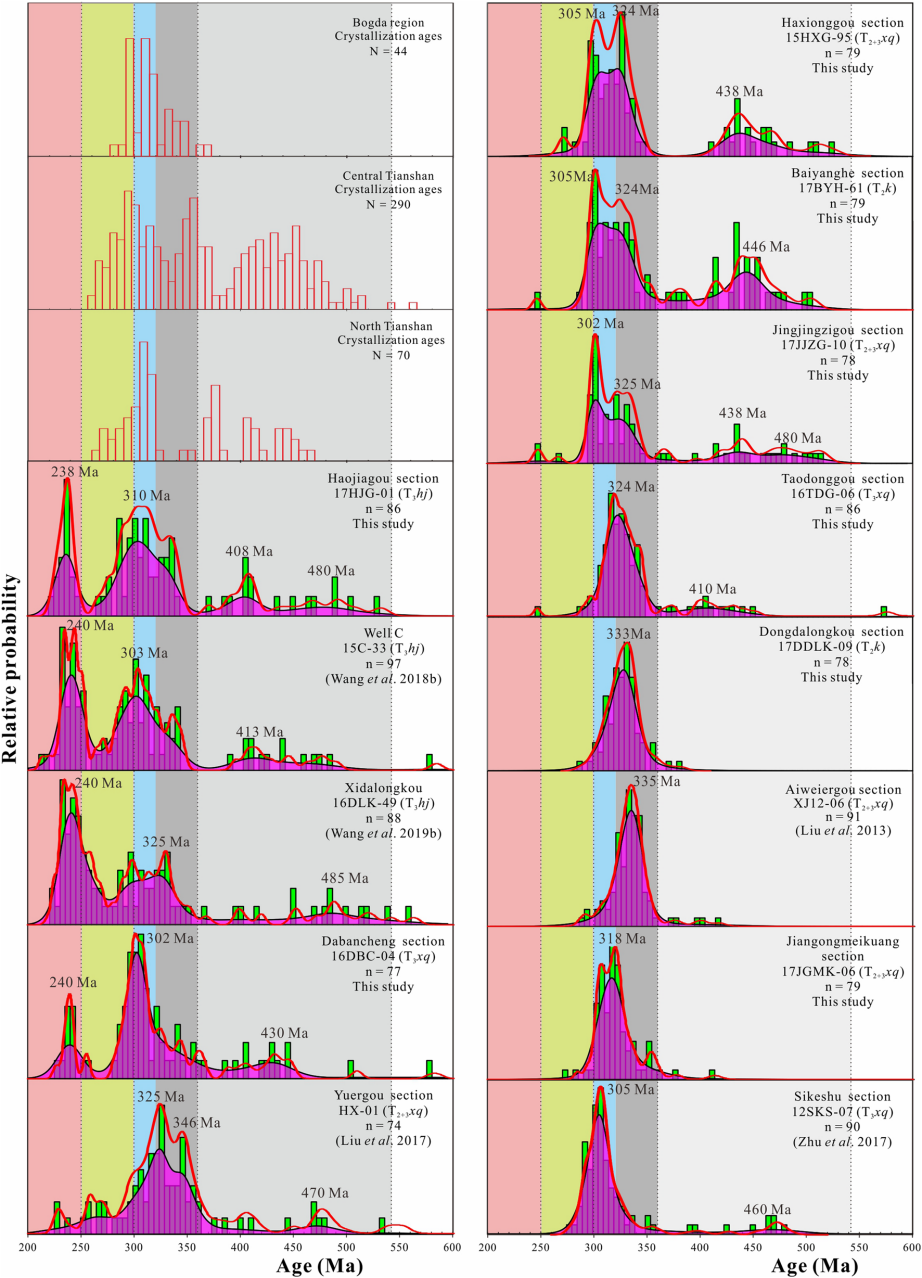
Combination of histograms, probability density plots, and kernel density estimates of detrital zircon grains derived from the Middle–Upper Triassic Xiaoquangou Group in different sections of the southern Junggar Basin. Histograms of crystallization ages from the STS–Tarim, Bogda Mountains, CTS, and NTS areas are also shown for comparison (see^17^ for data compilation).

Detrital zircon U–Pb ages for thirteen samples (12SKS-07, 17JGMK-06, XJ12-06, 17HJG-01, 15HXG-95, 17JJZG-10, 17BYH-61, 16DLK-49, 17DDLK-09, 16TDG-06, 16DBC-04, HX-01, and 15C-33) from various sections are given in Supplementary Information. The detrital zircon U–Pb concordia is plotted in Fig. 4. The combination of the histograms, probability density plots, and kernel density estimates of detrital zircon U–Pb ages are plotted in Fig. 5^22^. Most of the zircon grains exhibit oscillatory zoning, low luminescence, and high Th/U ratios (> 0.1), which are diagnostic of a magmatic origin^57^ (Supplementary Information). Zircon grains showing > 10% discordance are excluded from the interpretation (Supplementary Information).

According to previous works^12,16,17,58^, the pre-Triassic detrital zircon U–Pb ages for sedimentary rocks in the SJB and crystallization ages from the STS–Tarim, Bogda Mountains, CTS, and NTS areas are categorized into six populations, including the Precambrian (> 542 Ma), Cambrian–Devonian (541–361 Ma), Mississippian (360–320 Ma), Pennsylvanian (320–300 Ma), Permian (300–250 Ma), and Triassic populations (250–200 Ma; Fig. 5; Supplementary Information). The grouping of zircon ages in this study refers to this division, as displayed in Fig. 5. The sediments of the Middle–Upper Xiaoquangou Group from the Sikeshu (sample 12SKS-07) and Jiangongmeikuang (sample 17JGMK-06) sections predominantly contain Carboniferous zircon grains, which are mostly Pennsylvanian (320–300 Ma) in age (Fig. 5). In contrast, the sediments from the Aiweiergou (sample XJ12-06), Taodonggou (sample 16TDG-06) and Dongdalongkou (sample 17DDLK-09) sections show higher proportions of Mississippian zircon grains with ages in the range of 360–320 Ma (Fig. 5). The sediments from the Haxionggou section (sample 15HXG-95), Jingjingzigou section (sample 17JJZG-10), and Baiyanghe section (sample 17BYH-61) show two major peaks with age groups of both Pennsylvanian and Mississippian, and a minor peak with an age group of Cambrian–Devonian (541–361 Ma).

The detrital zircon U–Pb age patterns for samples of the Haojiagou section and Xidalongkou sections in the northern Bogda Mountains, and Dabancheng and Yuergou sections, and well C in the southern Bogda Mountains are similar, with principal age peaks and relative proportions of age groups comparable for various locations (Fig. 5). The detrital zircon grains of the samples from the well C and Haojiagou section yield age patterns dominated by Triassic populations, with subordinate populations of Pennsylvanian and Cambrian–Devonian ages (Fig. 5). The detrital zircon grains from the Xidalongkou section yield age patterns dominated by Triassic populations, with subordinate populations of Mississippian and Cambrian–Devonian ages (Fig. 5). The detrital zircon grains from the Dabancheng section display a relatively lower proportion of the Triassic age group, yielding age patterns dominated by Pennsylvanian, accompanied by subordinate Triassic and Cambrian–Devonian populations (Fig. 5). The detrital zircon age group for the Yuergou section sample is dominated by Mississippian ages, with minor Cambrian–Devonian constituents (Fig. 5).

### Zircon trace elements

**Figure 6:**
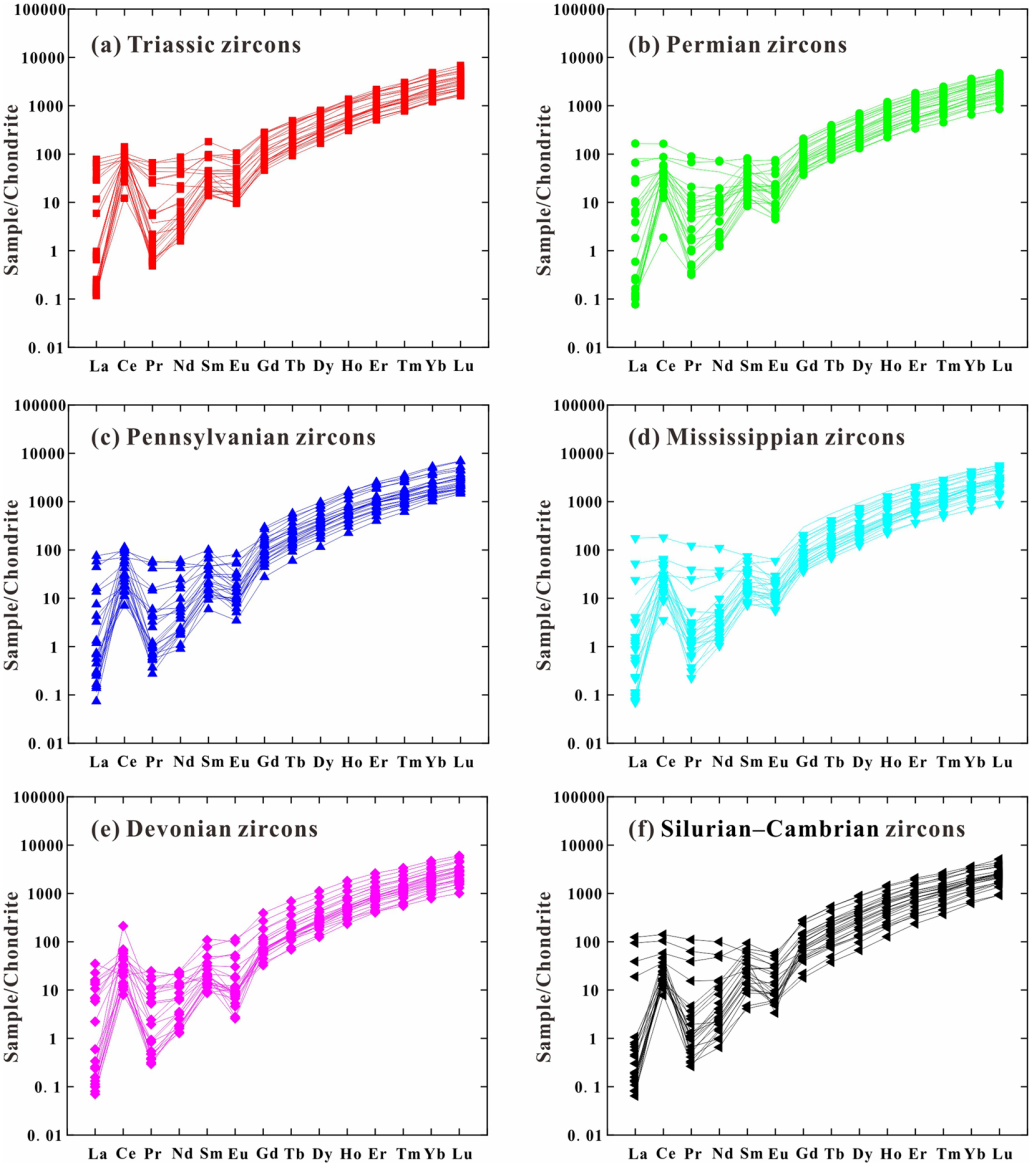
Chondrite-normalized REE patterns for the **(a)** Triassic, **(b)** Permian, **(c)** Pennsylvanian, **(d)** Mississippian, **(e)** Devonian, and **(f)** Silurian–Cambrian zircon grains. Chondrite values are from^59^.

**Figure 7:**
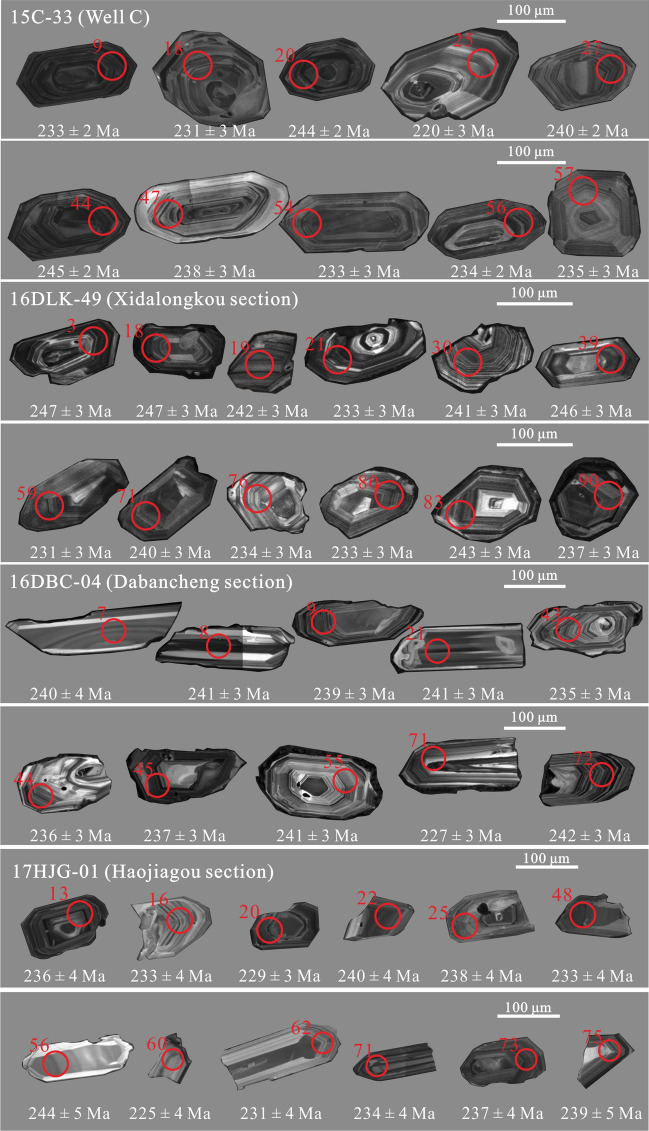
Representative cathodoluminescence (CL) images of the Triassic zircon grains.

**Figure 8:**
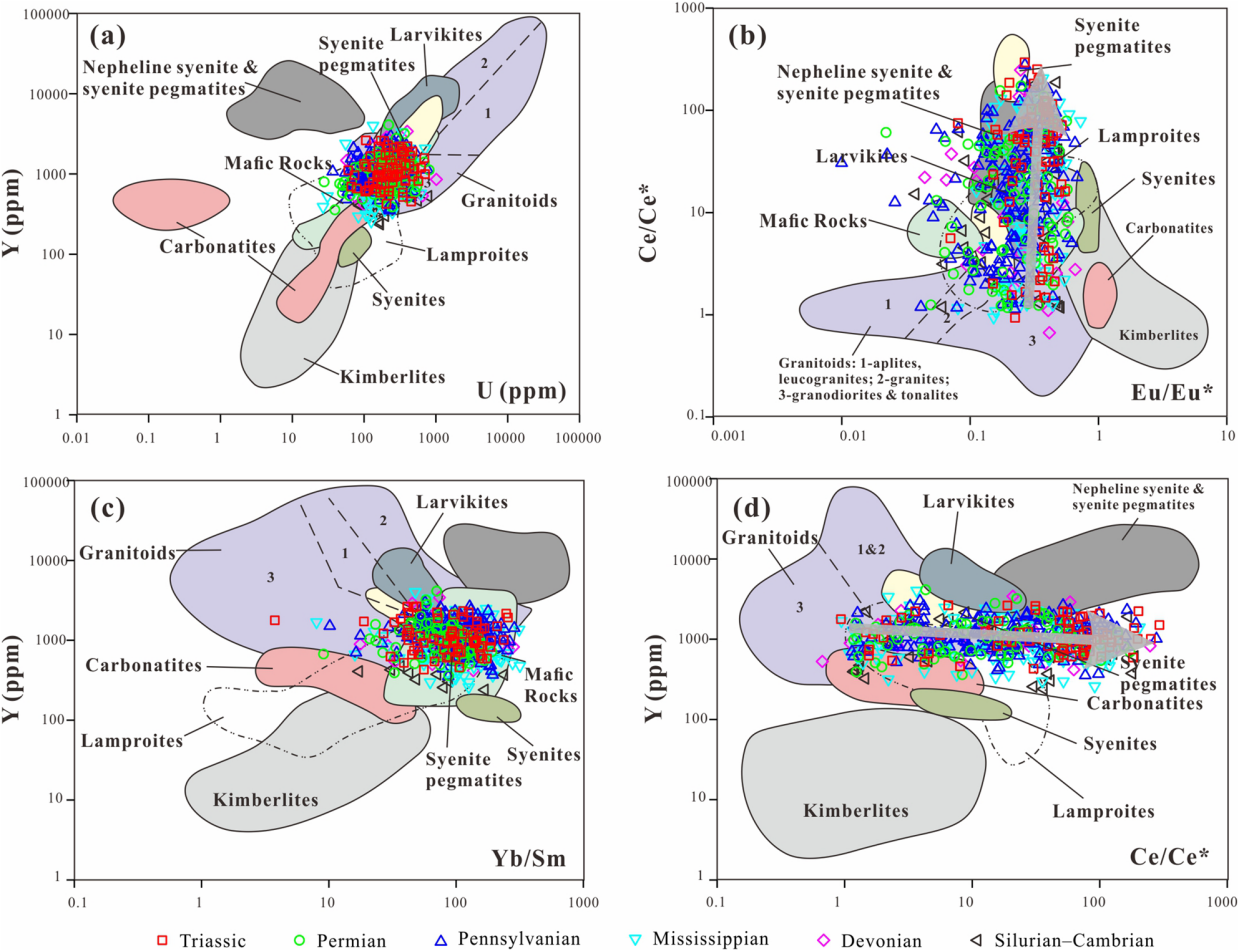
Zircon compositions of **(a)** U–Y, **(b)** Eu/Eu^*^–Ce/Ce^*^, **(c)** Yb/Sm–Y, and **(d)** Ce/Ce^*^–Y diagrams used as discriminants for different source rock types (modified after^3^).

The results from the geochemical analysis of the detrital zircon grains are listed in Supplementary Information. Zircon shows a wide range of rare earth element (REE) concentrations (218–10,178 ppm, mostly 400–900 ppm), with average concentrations significantly above those for chondrite^59^ (2.65 ppm). Most of the zircon grains, irrespective of the age groups, are depleted in light REEs and enriched in heavy REEs, as displayed in the chondrite plots^59^ (Fig. 6). The majority of the analyzed zircon grains exhibit negative Eu and positive Ce anomalies (Fig. 6; Supplementary Information; Eu/Eu^*^ = 0.01–0.72; Ce/Ce^*^ = 1.1–357.8), oscillatory zoning, low luminescence, and high Th/U values (> 0.1), which are diagnostic of a magmatic origin^2,3,57^(Fig. 7). The Triassic zircon grains (Fig. 6a) show REE concentrations and chondrite-normalized patterns similar to those of the Cambrian–Permian zircon grains (Fig. 6b–f; Supplementary Information). On the zircon U–Y, Eu/Eu*–Ce/Ce*, Yb/Sm–Y, and Ce/Ce*–Y diagrams, most of the zircon grains with age groups of Cambrian–Devonian, Mississippian, Pennsylvanian and Permian populations plot in granitoid (acidic rock) areas^3^ (Fig. 8). Zircon with Triassic ages shows features similar to those of granitoid (acidic rock) and syenite (intermediate rock)^3^ (Fig. 8).

### Multidimensional scaling

**Figure 9:**
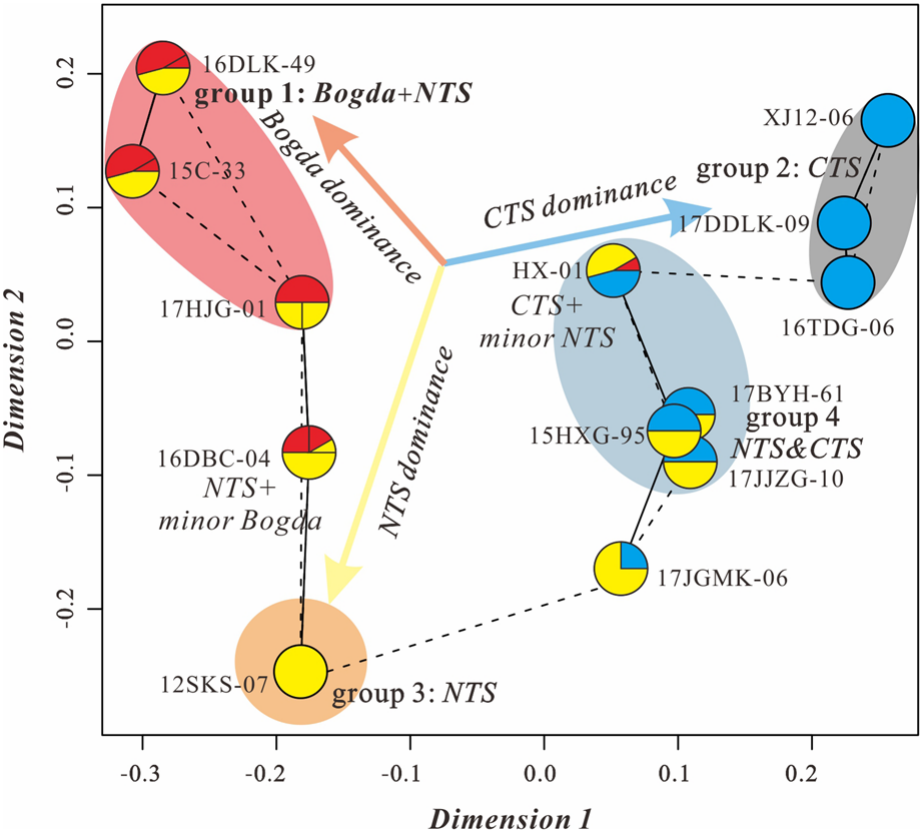
Multidimensional scaling plots (MDS) for detrital zircon U–Pb data of the samples from the Middle–Upper Triassic Xiaoquangou Group in different measured sections of the southern Junggar Basin^22^. Solid and dashed lines represent the closest and second closest neighbors, respectively. NTS, North Tianshan; CTS, Central Tianshan; Bogda, Bogda Mountains.

The detrital zircon U–Pb age probability and multidimensional scaling (MDS) plots were used in conjunction to establish a Middle–Late Triassic source-to-sink system of the SJB^22^ (Figs. 5 and 9). In the MDS plot, 13 samples from the Middle–Upper Triassic Xiaoquangou Group in the SJB show distinct differences that generate four groups according to their detrital zircon age distributions (Fig. 9). The similar samples cluster closely together relative to their similar provenances, while the dissimilar samples plot far apart revealing that they have different source areas (Fig. 9).

Samples 15C-33, 16DLK-49 and 17HJG-01 (group 1) from the well C and Xidalongkou and Haojiagou sections differ significantly from the others (Fig. 9). Samples XJ12-06, 16TDG-06, and 17DDLK-09 (group 2) from the Aiweiergou, Taodonggou and Dongdalongkou sections are assigned similar sediment sources owing to their close links in the MDS plot (Fig. 9). Samples 12SKS-07, 17JGMK-06, and 16DBC-04 (group 3) from the Sikeshu, Jiangongmeikuang and Dabancheng sections generally display a limited age variation and show proximity in the MDS plot (Fig. 9). Samples 15HXG-95, 17JJZG-10, 17BYH-61, and HX-01 (group 4) from the Haxionggou, Jingjingzigou, Baiyanghe and Yuergou sections show a mixed age variation and have proximity in the MDS plot (Fig. 9).

## Discussion

### Provenance of Triassic detrital zircon in the southern Junggar Basin

Considering that paleocurrents are mainly northward-directed in the southern Junggar Basin, the potential source areas are situated mainly to the south in the Tianshan area^7,8,10,12,16,17,41,52^. Based on the comparison of the detrital zircon U–Pb age distribution to the reported crystallization ages of intermediate–acidic igneous rocks from the NTS, CTS, and Bogda Mountains, the Cambrian–Devonian and Mississippian age group zircon grains are derived from the CTS, and the Pennsylvanian age group zircon grains are likely sourced from the NTS^16,17^ (Fig. 5). In particular, constraining the provenance of Triassic detrital zircon is difficult because reports suggest that there is relatively sparse direct field evidence of Triassic magmatic activity in the Junggar Basin and adjacent areas.

Although limited field evidence of Triassic magmatic or volcanic activity exists, numerous studies have indicated that Triassic magmatism or volcanism is extensively distributed both in the Junggar Basin and in the surrounding areas. In the Altai Mountains, the Late Triassic magmatism (zircon U–Pb age of 222 ± 2 Ma) was found based on the Huoremudeke granitic pluton^26^. In the Bogda Mountains, thin amygdaloidal andesites with no precise ages are interbedded in the sequences of Middle Triassic clastic rocks of the Kelamayi Formation^25^. In the Eastern Tianshan (south of the Turpan–Hami Basin), Greene et al.^38^ reported a granitoid pluton with a zircon U–Pb age of 229 ± 11 Ma in the Barkol Tagh. In addition, many Triassic detrital zircon grains with ages of 250–200 Ma are reported from sediments of the West Junggar Basin, southern Junggar Basin, Bogda Mountains, central Turpan–Hami Basin and central Junggar Basin^10,16,17,60–62^. Theoretically, the Bogda Mountains, Altai Mountains, West Junggar, NTS, CTS, and STS could be the source area for sediments included the Triassic detrital zircon grains.

To constrain the provenance of Triassic detrital zircon grains in the SJB, detrital zircon U–Pb geochronological, sandstone petrological, sedimentary geochemical, and paleocurrent data were integrated. First, analysis of the depositional environment reveals that the Junggar Basin contains mainly lacustrine facies in the Middle–Late Triassic strata^16,17,25,39^ (Fig. 2), and sedimentary rocks of many areas have high sediment textural maturity. However, in the Bogda Mountains, sediments of the Middle–Upper Triassic Xiaoquangou Group show low sediment textural maturity with angular to subangular lithic fragments and detrital zircon grains^16^ (Fig. 7), suggesting a proximal source area. Second, paleocurrent observations show that the drainage system is perpendicular to the Bogda Mountains, with northward-directed paleocurrents in the northern Bogda Mountains, and southwestward-directed paleocurrents in the southern Bogda Mountains (Fig. 3), suggesting that the nearest Bogda Mountains are probably the source area. Third, sediments from the Middle–Upper Triassic Xiaoquangou Group have a high sediment component maturity with high quartz contents, some sedimentary lithic fragments and low feldspar grains, and exhibit moderate weathering and sedimentary recycling signatures^17^, suggesting that the detritus is probably sourced from recycling of older sediments in the Bogda Mountains. Thus, the Permian or Early Triassic older clastic rocks in the Bogda Mountains have been unroofed and denuded. Fourth, detrital zircon U–Pb dating results for 13 samples reveal the dominance of Triassic detrital zircon grains in sediments from the Haojiagou and Xidalongkou sections (northern Bogda Mountains), with subordinate populations in sediments from the well C, and Dabancheng and Yuergou sections (southern Bogda Mountains), and no or minor amounts appear in sediments from other areas like the NTS and CTS (e.g., Sikeshu, Jiangongmeikuang and Aiweiergou sections; Fig. 5). The Triassic detrital zircon grains occur mainly in sediments from the Bogda Mountains and are absent in the NTS and CTS areas, suggesting that the Triassic detrital zircon grains were probably derived from the Bogda Mountains. Fifth, the proportion of Triassic detrital zircon grains is centered in the Bogda Mountains and decreases to the periphery. In the southward direction, the proportion of Triassic detrital zircon grains decreases steadily from the well C, and Haojiagou and Xidalongkou sections to the south of the Dabancheng, Taodonggou and Yuergou sections, and further to the Aiweiergou and Jiangongmeikuang sections (Figs. 2 and 5). In the eastward direction, the proportion of Triassic detrital zircon grains decreases steadily from the Xidalongkou and Baiyanghe sections to the east of the Dongdalongkou section (Figs. 2 and 5). This suggests that the Triassic detrital zircon grains are derived from the Bogda Mountains and are restricted to the Urumqi–Jimusaer area and probably the Haojiagou–well C–Xidalongkou areas. In summary, the provenance of Triassic detrital zircon grains from sediments of the Xiaoquangou Group in the SJB is the nearest Bogda Mountains. Note that not all the Triassic zircon grains are derived from the Bogda Mountains; for example, the Balikun area may have received material from the Barkol Tagh (Fig. 2).

In addition, the trace element composition of detrital zircon grains is sensitive to the source rock type^2,3,57,63^. The variations in representative elements and ratios of REEs for zircon grains indicate that the Triassic detrital zircon originated from intermediate–acidic igneous rocks, with probable dominance of intermediate igneous rocks^2,3^ (Fig. 8). We attribute the Triassic volcanic rocks are likely interlayered in the Middle–Upper Triassic Xiaoquangou Group of the Bogda Mountains, as suggested by BGMRXUAR^25^.

This study corroborates the utility of the comparison of detrital zircon U–Pb age distributions for identifying source locations, especially when the potential source areas lack direct zircon field evidence (e.g.^11,22^). It also confirms that this provenance analysis tool is an effective approach for deciphering source characteristics in deep-time source-to-sink system.

### Source-to-sink systems of the SJB in the Middle–Late Triassic

**Figure 10:**
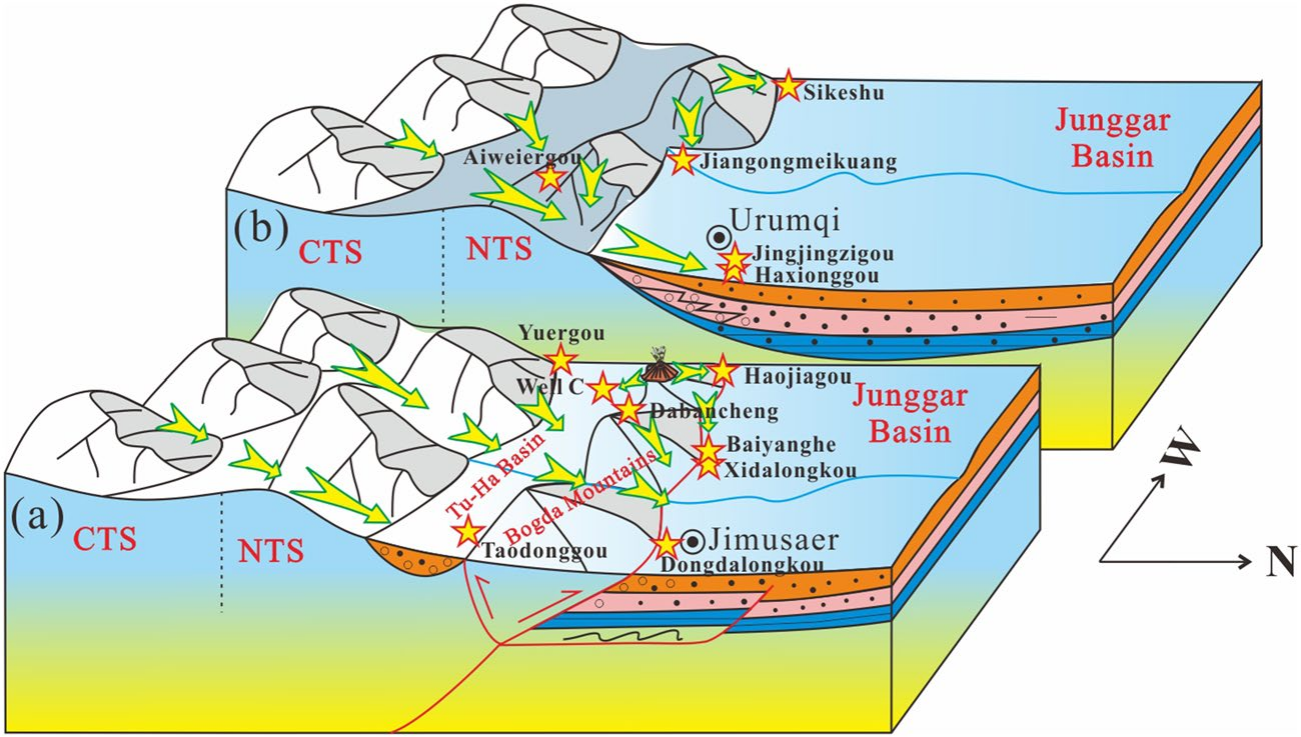
Schematic 3D diagram demonstrating the source-to-sink system of the **(a)** eastern part (Bogda mountains area) and **(b)** western part (front of the NTS) of the southern Junggar Basin in the Middle–Late Triassic. *NTS* North Tianshan, *CTS* Central Tianshan.

The systematic analysis of detrital zircon grains preserved in the Xiaoquangou Group allowed us to discriminate their source areas and establish the source-to-sink systematics in the Middle–Late Triassic for the SJB. The provenance analysis of thirteen samples partitions the SJB and adjacent areas into four groups (Fig. 10), which is consistent with groups from the MDS plot (Fig. 9). The four groups are derived from at least four source-to-sink systems in the Middle–Late Triassic time (Fig. 10).

Group 1 from the well C, and Xidalongkou and Haojiagou sections has high contents of Triassic zircon grains (Figs. 5 and 9), indicating a predominant contribution from the Bogda Mountains. Moreover, group 1 contains subordinate Carboniferous and Cambrian–Devonian zircon grains, revealing that the NTS and/or CTS also provided sediments to the northern Bogda Mountains (Figs. 5 and 9). That is, the provenance of sediments for group 1 is primarily attributed to the Bogda Mountains, with secondary contributions from the NTS and/or CTS. Group 2 from the Aiweiergou, Taodonggou and Dongdalongkou sections includes lots of Mississippian and Cambrian–Devonian zircon grains, suggesting that the CTS are likely major sources (Figs. 5 and 9). Group 3 from the Sikeshu section displays a limited age variation that is dominated by Pennsylvanian (Fig. 5), combined with the compositionally and texturally immature detrital grains, indicating that group 3 is originated from the igneous rocks of the NTS with zircon U–Pb ages of 320–300 Ma (Fig. 5). Group 4 from the Haxionggou, Jingjingzigou, and Baiyanghe sections shows a mixed age variation of Pennsylvanian (320–300 Ma) and Mississippian (360–320 Ma), revealing that the major provenance of group 4 is from the NTS and CTS (Figs. 5 and 9). Summarising, the Bogda Mountains, CTS, and NTS appear to be the dominant sources for group 1, group 2, and group 3, respectively (Fig. 9); a mixture of the NTS and CTS appears to be the dominant source for group 4 (Fig. 9).

Although the source areas of the SJB include the Bogda Mountains, NTS, and CTS in the Middle–Late Triassic, the western (front of the NTS) and eastern (Bogda Mountain areas) parts of the SJB exhibit inconsistencies in their provenance patterns. In the Bogda Mountains, the provenance is attributed to the Bogda Mountains itself and the NTS (Figs. 9 and 10). Of these, the Bogda source mainly provides syndepositional and recycled sediments. In the NTS areas, the NTS piedmont (e.g., Sikeshu and Jiangongmeikuang sections) received material mainly from the NTS, with minor input from the CTS and no input from the Bogda Mountains. In the transition zone of the western and eastern parts of the SJB, such as the Haojiagou section area, the basin received material from both the NTS and Bogda Mountains. In conclusion, the NTS accounts primarily for the provenance of the western part of the SJB (front of the NTS), while sediments from the eastern part of the SJB (Bogda Mountains areas) are principally derived from the Bogda Mountains (Figs. 2, 5, 9, 10).

### Implication for the tectonic evolution of the southern Central Asian Orogenic Belt

**Figure 11:**
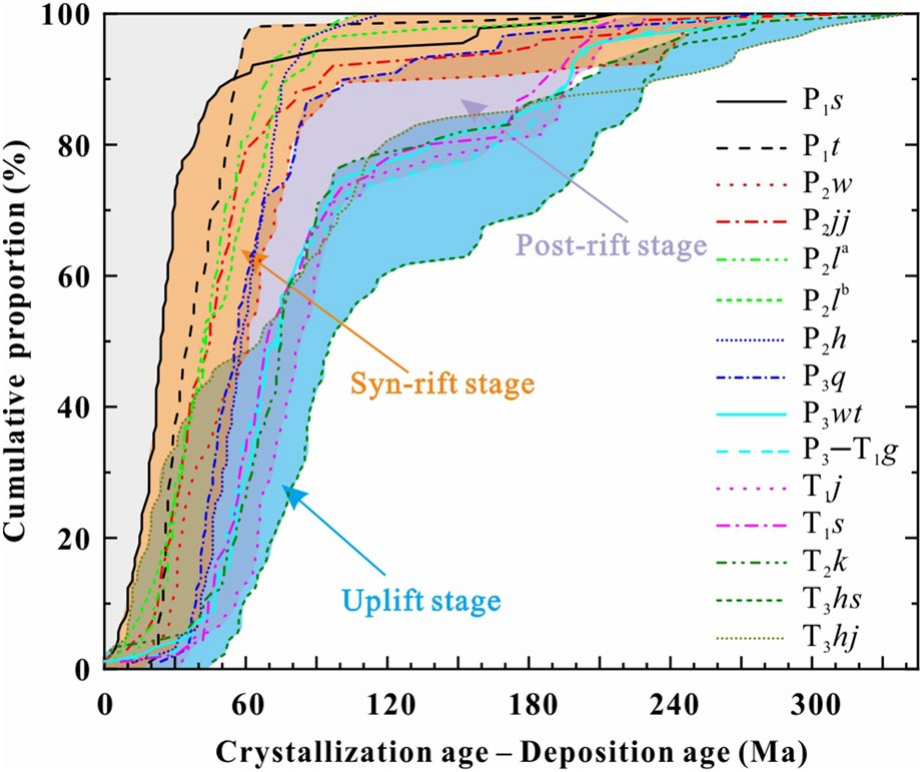
Cumulative frequency curves of the lag time (crystallization age–depositional age) for the Cisuralian Shirenzigou Formation to the Upper Triassic Haojiagou Formation. The data and tectonic evolution stages are from^16,17^. P_1_*s*, Shirenzigou Formation; P_1_*t*, Tashikula Formation; P_2_*w*, Wulapo Formation; P_2_*jj*, Jingjingzigou Formation; P_2_*l*^a^, lower Lucaogou Formation; P_2_*l*^b^, upper Lucaogou Formation; P_2_*h*, Hongyanchi Formation; P_3_*q*, Quanzijie Formation; P_3_*wt*, Wutonggou Formation; P_3_–T_1_*g*, Guodikeng Formation; T_1_*j*, Jiucaiyuanzi Formation; T_1_*s*, Shaofanggou Formation; T_2_*k*, Kelamayi Formation; T_3_*hs*, Huangshanjie Formation; T_3_*hj*, Haojiagou Formation.

The Middle–Upper Triassic Xiaoquangou Group in well C, and Haojiagou and Xidalongkou sections yields a complex age population with many syndepositional Triassic detrital zircon grains from the Bogda Mountains (Fig. 5), indicating the initiation of sediment supply from the Bogda Mountains in the same area. We attribute this abrupt change to the initial uplift of the Bogda Mountains^17^ (referred to as the uplift stage). Moreover, the Xiaoquangou Group in the Haojiagou and Xidalongkou sections contains lithic fragments^16,40^, with sediments characterized by moderate chemical weathering and sedimentary recycling^17^. This suggests that the Bogda Mountains provided syndepositional volcanics and recycled siliciclastics to the basin. In addition, according to the tectonic cycle identified by previous works^17,33,35^ (the SJB entered in the tectonic evolution of post-orogenic since the Pennsylvanian, of these, the Pennsylvanian to Guadalupian referred to as the syn-rift stage which characterized by faulting, the Lopingian to Early Triassic referred to as the post-rift stage which is characterized by depression with little faulting, and the Middle–Late Triassic referred to as the uplift stage due to the uplift of the Bogda Mountains), we draw cumulative frequency curves of the lag time^5^ (lag time = crystallization age – deposition age; Fig. 11) for the Cisuralian Shirenzigou Formation to the Upper Triassic Haojiagou Formation. In the cumulative frequency curves of the lag time^5^ (Fig. 11), the sediments of the uplift stage show a near “bimodal lag time”, including low and high lag time groups, compared to mainly low lag time groups for syn-rift and post-rift sediments^5^ (Fig. 11). The high lag time sediments probably arose from sedimentary recycling, while the low lag time sediments are attributed to syndepositional magmatic activity.

Although the proportion of Cambrian–Devonian, Mississippian, Pennsylvanian, and Permian populations diminishes, these populations do not vanish compared to the underlying units^16,17^. This implies that both the NTS and CTS continued providing sediments for sedimentation in the Bogda Mountains. This is further evidenced by the detrital zircon U–Pb age cumulative frequency curves, probability plot and kernel density estimates, which exhibit zircon age groups such as syn-rift and post-rift sediments (Figs. 5 and 11). We attribute this to the Late Triassic uplift of the Bogda Mountains, but the relatively smooth relief of its topography allowed the scouring by rivers and consequently the supply of detritus from the NTS and CTS (Figs. 2 and 10).

On the other hand, the data of this study has suggests that the Triassic magmatism or volcanism is extensively distributed both in the Junggar Basin and in the surrounding areas. The available thermal data including zircon (U–Th)/He, biotite ^40^Ar–^39^Ar, titanite fission track, and apatite fission track dates suggest that the Central Asian has undergone extensive cooling and exhumation in the Triassic^64^. Summarizing we can show that the Triassic event, which has not received much attention, is widespread in the southern CAOB. The Triassic event in the southern CAOB coincides with the Qiangtang–Eurasia collision^65^. Thus, we suggest that the Qiangtang Block collision with Eurasia probably induced fault reactivations and basement exhumation within the southern CAOB^64^. The Late Triassic uplift of the Bogda Mountains resulted from the far field effect of the Qiangtang–Eurasia collision (e.g.^42,66^). We conclude the Triassic event is a tectonic reactivation (induced by Qiangtang–Eurasia collision) within the post-orogenic tectonic evolution of the southern CAOB.

## Conclusions

Comparison of detrital zircon U–Pb age distributions enabled the constraining of the location of Triassic volcanic activity in the SJB area. The comparison of the new detrital zircon U–Pb age dataset for the Middle–Upper Triassic Xiaoquangou Group of the SJB shows that Triassic volcanic activity occurred mainly in the Urumqi–Jimusaer area. The REE abundance of zircon suggests that Triassic volcanism resulted in mainly intermediate–acidic rocks. The geochronology data combined with provenance analysis indicate that provenance varies for different areas in the SJB, resulting in four sink areas. The Bogda Mountains started to supply sediments in the Late Triassic, marking the initial uplift of the Bogda Mountains. This study proves the effectiveness of the comparison of detrital zircon U–Pb age distributions for inferring source signatures in deep-time source-to-sink system.

## Supplementary Information

Supplementary Tables.

## Data Availability

All data generated or analysed during this study are included in this published article and its Supplementary Information files.
